# Supplementary material for: Human iPS-derived pre-epicardial cells direct cardiomyocyte aggregation expansion and organization in vitro
Source: Nat Commun. 2021 Aug 17;12:4997. doi: 10.1038/s41467-021-24921-z (PMC8370973; doi:10.1038/s41467-021-24921-z)
Supplement: Supplementary file 1 — Supplementary Information [file 41467_2021_24921_MOESM1_ESM.pdf]

Supplementary Information

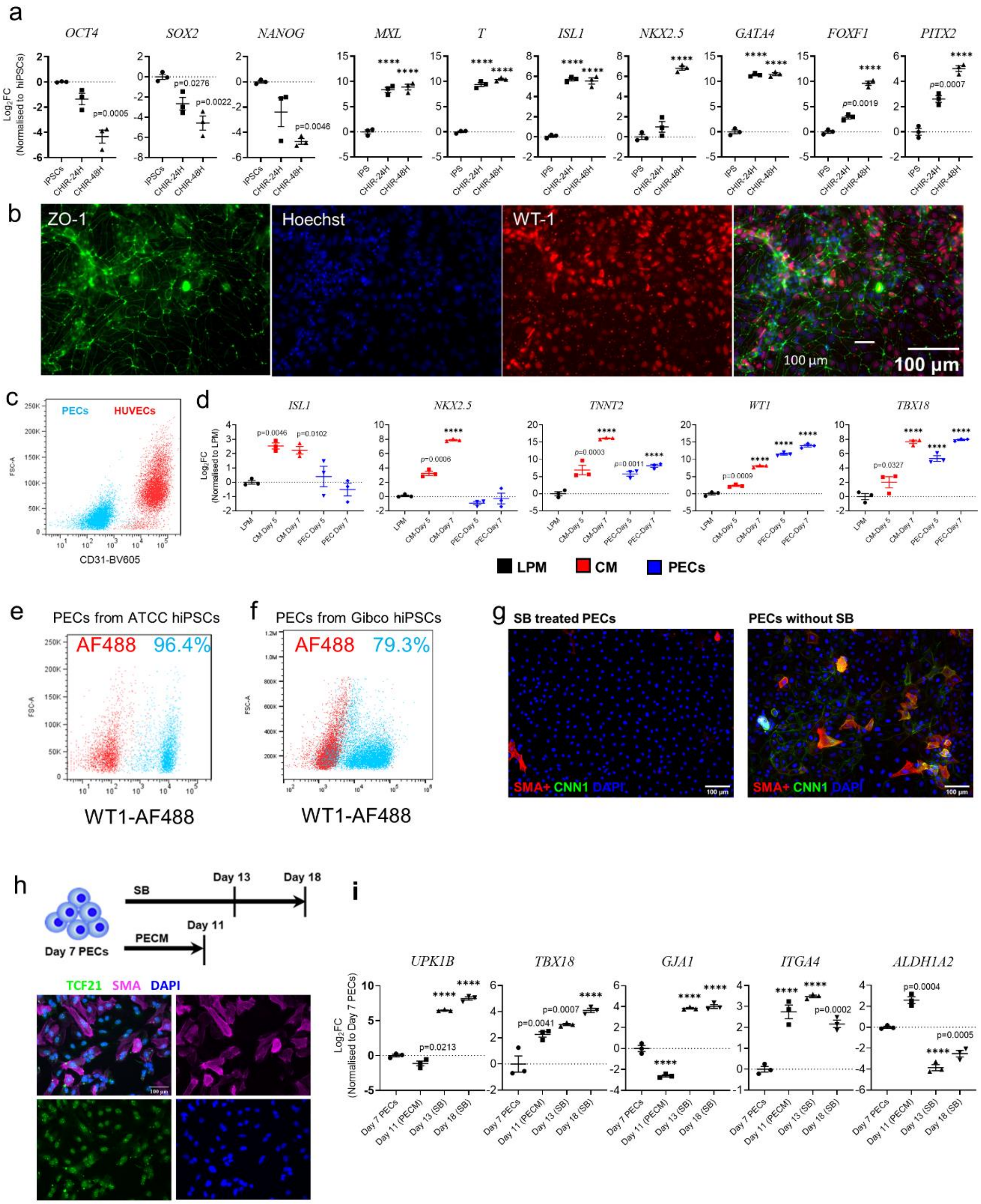

Supplementary Fig 1: Stage-Characterization of PEC Differentiation

a RTPCR Characterization of LPM at day 3 following 24 h and 48 h of CHIR99021 treatment, pluripotency markers (i.e. *OCT4*, *SOX2* and *NANOG*), Primitive streak markers (i.e. *MXL* and *T*), cardiac progenitor markers (i.e. *GATA4, ISL1*, and *Nkx2.5*) and lateral plate mesoderm marker (i.e. *PITX2* and *FOXF1*) expressions at day 3, with fold change normalized to hiPSC expression at day 0. Data presented are mean $\log_2$ fold change ±SEM (n=3 independent differentiation). One-way ANOVA with Dunnett's post-hoc test was used versus expression in iPSCs. *P* value presented on the graph corresponds to

the respective column. ****p<0.001 versus hiPSCs. **b** Representative immunofluorescent images of day 7 PECs from three independent differentiation (n=3), stained for WT1 and ZO1. Scale =100 µm. **c** Representative FACS plot showing CD31 expression in PECs (Blue) and HUVEC cells (Red) (experiment repeated 3 times, FACS n=1). **d** RTPCR characterization of differentiating of PECs and CMs from LPM at day 5 and day 7. Data presented are mean $\log_2$ fold change ± SEM normalized to LPM (n=3 independent differentiation). One-way ANOVA with Dunnett's post-hoc test was used versus expression in iPSCs. *P* value presented on the graph corresponds to the respective column. ****p<0.001 versus LPM. **e-f** FACS plot of day 7 PECs differentiated from hiPSC lines obtained from ATCC (F) and Gibco (G). Red are secondary stained population and blue are WT1-stained population. **g** Representative images of PEC in maintenance culture (n=3 independent differentiation). Immunostaining of PECs maintained in SB-supplemented medium (left) and basal medium (right). Scale bar=100µm. Red, smooth muscle actin (SMA); Green, calponin (CNN1); Blue, DAPI. **h** PEC maintenance with SB-supplemented medium and PEC medium (PECM). Schematic illustrates the timeline for sampling PECs after culturing with SB-supplemented medium and PECM for RT-PCR analysis. Immunostaining of TCF21+ (Green) and SMA+ (Magenta) PECs after 6 days in PECM culture. Scale = 100 µm. The expression of epicardial-related markers after culturing in PECM (at day 11) and SB supplemented medium (at day 13 and 18) relative to the expression in freshly differentiated PECs at day 7, were presented in mean $\log_2$ fold change ±SEM (n=3 independent experiments). One-way ANOVA with Dunnett's post-hoc test was used versus expression in day 7 PECs. *P* value presented on the graph corresponds to the respective column. ****p<0.001 versus day 7 PECs.

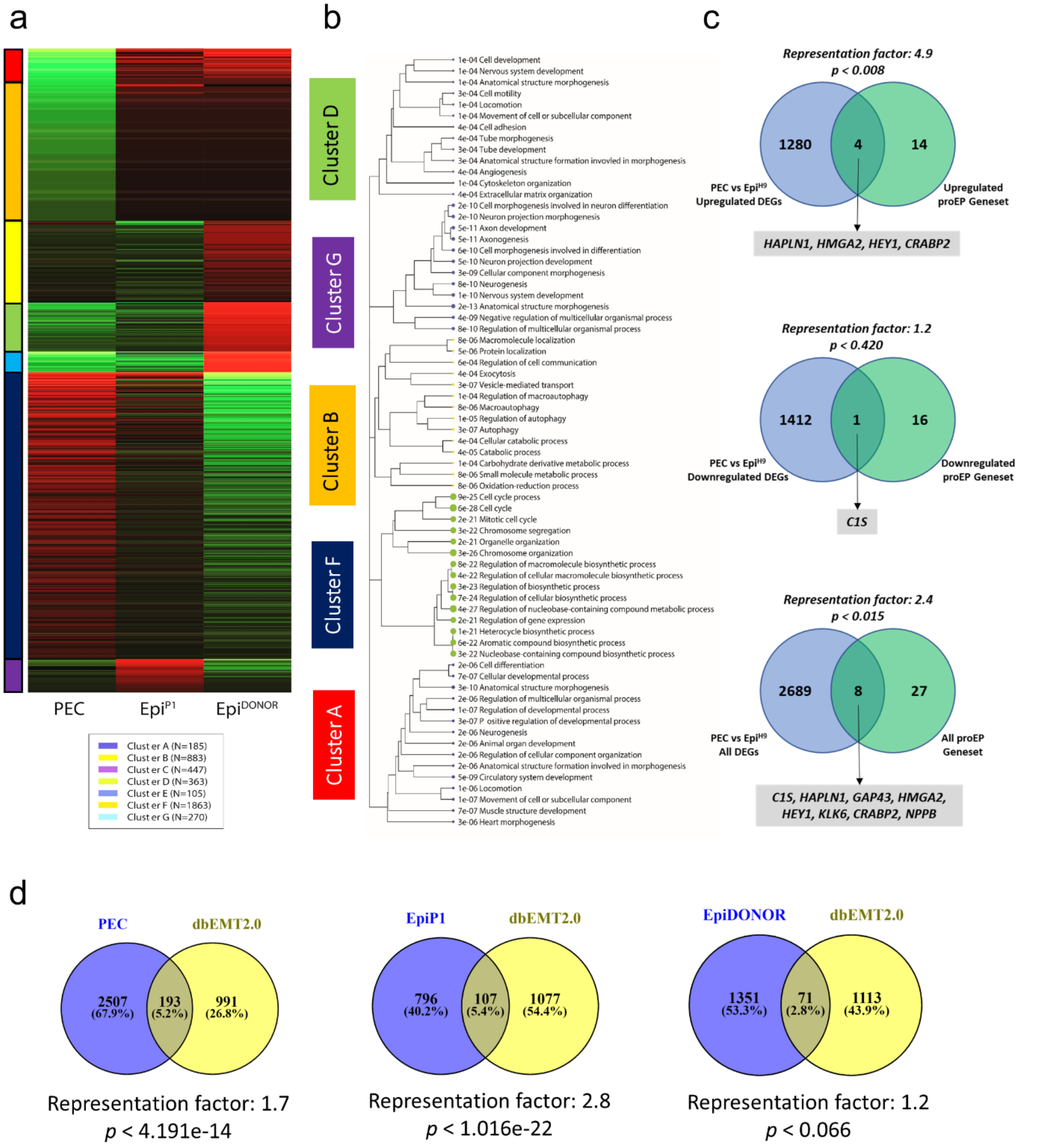

Supplementary Figure 2: RNA Sequencing analysis of PEC, EpiP1 and EpiDONOR.
a K-means clustering of differentially expressed genes (DEGs) from EpiP1(n=2) vs EpiH9 (n=3), PEC (n=3) vs EpiH9 and EpiDONOR vs EpiSC comparisons. **b** Functional clustering of enriched gene ontology terms for biological processes for clusters A, B, D, F and G. **c** Enrichment analysis using the exact hypergeometric probability test on the DEGs lists for PEC and EpiP1 with the proepicardial (proEP) geneset extracted from the Cui et al. (2019) study 21. **d** Exact hypergeometric probability for overlapped differentially expressed genes (DEGs) and dbEMT2.0 curated genes. Individual list of DEGs from PEC vs EpiH9 (PEC, 2700 DEGs), EpiP1 vs EpiH9 (EpiP1, 903 DEGs) and EpiDONOR vs EpiSC (EpiDONOR, 1422 DEGs) analyses were used to compare with 1184 dbEMT2.0 curated genes to determine the statistical significance of the overlap between two groups of genes. All the unique genes found in both studies (28395 genes) were used as the background for the analysis.

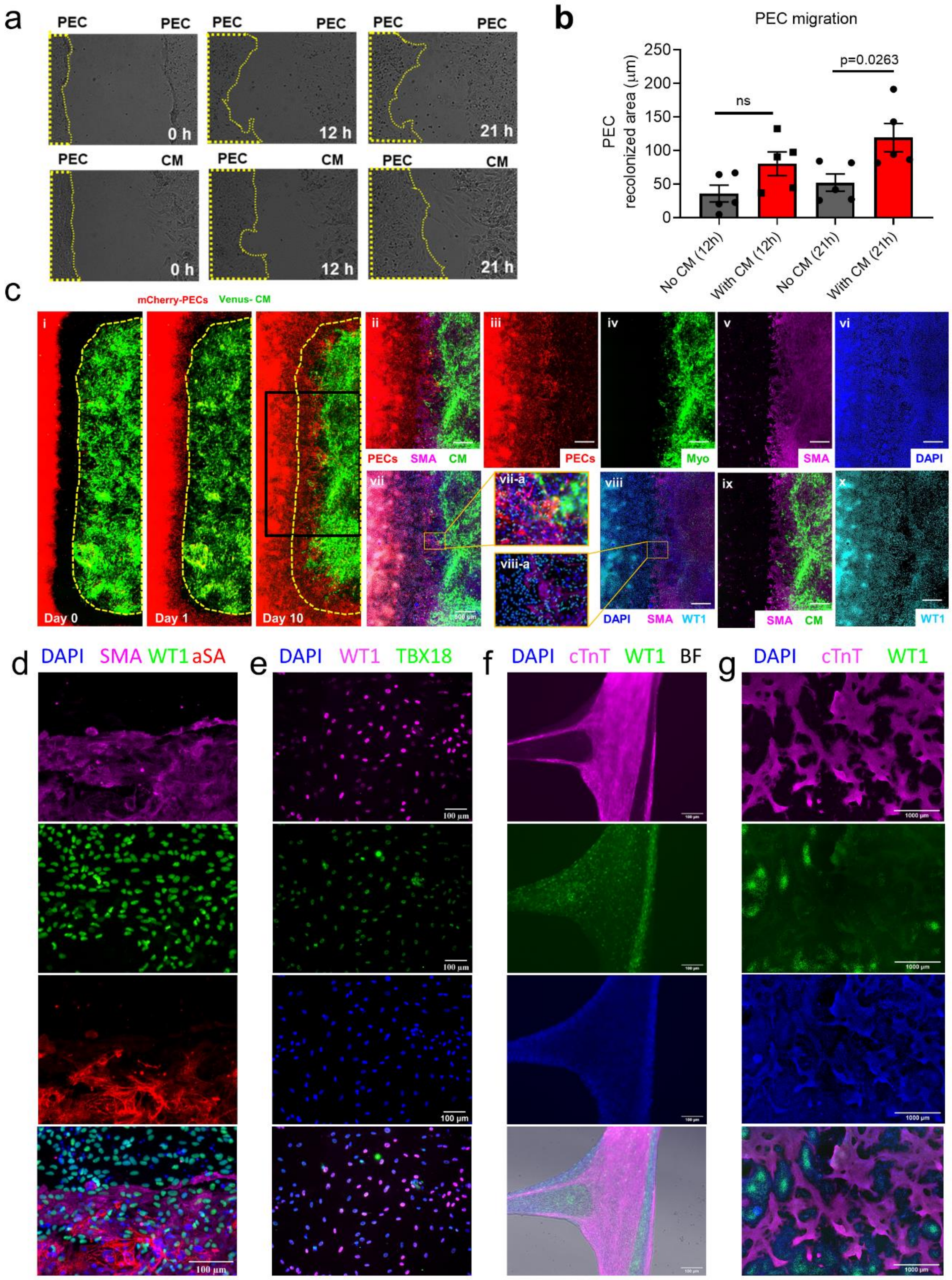

Supplementary Figure 3: PEC migration and its effect on CM in Coculture.

a Representative image of the migration of mitomycin treated PECs (yellow dotted box) to the gap was imaged at 0h, 12h and 21 h with or without CM in co-culture. Data presented are mean ± SEM, n=3 independent experiments. **b** PEC recolonized area was measured after 12h and 21 h with or without CM. Data presented are mean ± SEM, n=5 independent experiments using PECs from different differentiation batches. Unpaired, two-tailed student T-test was used for PEC recolonized area in no CMs vs with CMs at the same time point (12h or 21h). **c** Representative images of mCherry-PECs (red) and Venus-CMs (green) in co-culture at (i) day 0, day 1 and day 10 from three independent experiments. Immunofluorescence images of area-of-interest (black box) staining CM and PECs for WT1 and SMA after 10 days (ii-x). **d** Representative images of PECs

at CM border staining for WT1 (green), smooth muscle actin (magenta) and alpha sarcomeric actin (red). Nuclei were stained with DAPI (blue). Scale: 100 µm, n=3 independent experiments. **e** Representative images from three independent experiments of the re-isolated PECs from 6 days CM coculture showed WT1 (magenta) and TBX18 (green) expressions. **f-g** Representative images of the PEC/CM co-differentiation culture from three independent experiments showed WT1 (Green) and cTnT (magenta) expressions. BF, bright field

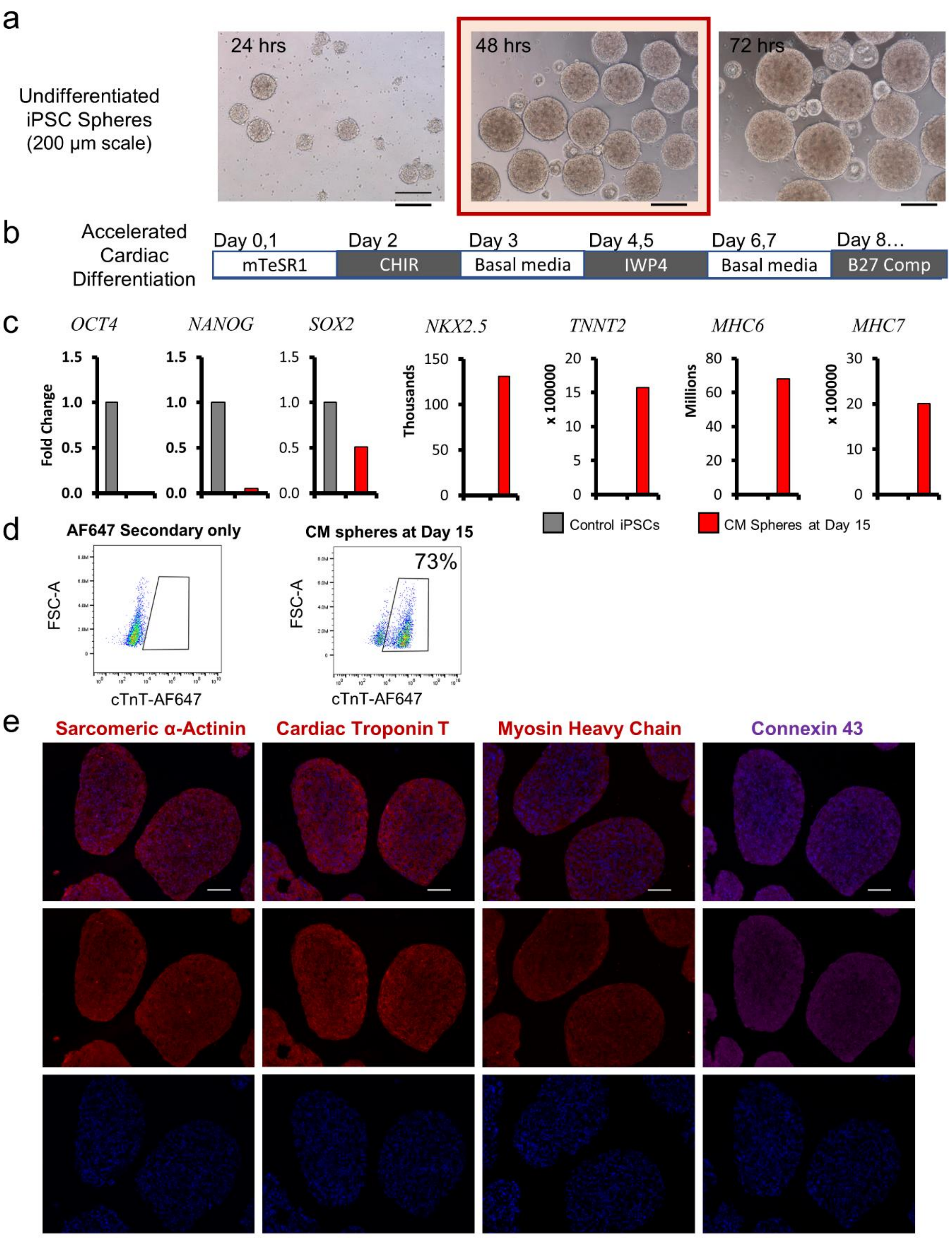

Supplementary Figure 4: Characterization of CM-Spheres.
a Sphere formation of undifferentiated human iPS cells in spinner flask culture at 24, 48, and 72 h at 10x magnification. Spheres at 48 h were used for cardiac differentiation. Images were the representation obtained from three different differentiation experiments Scale, 200 µm. **b** Schematic depicting the timeline for cardiac differentiation of iPS spheres. **c** Gene expression of CM-spheres (day 15 after onset of differentiation) versus control iPS cells (i), and **d** representative flow cytometry results indicating day 15 CM-spheres are 73% cTnT+ (n=1). **e** Histological analysis of day 15 CM-spheres, staining positive for a panel of cardiac-specific markers. Scale, 100 µm.

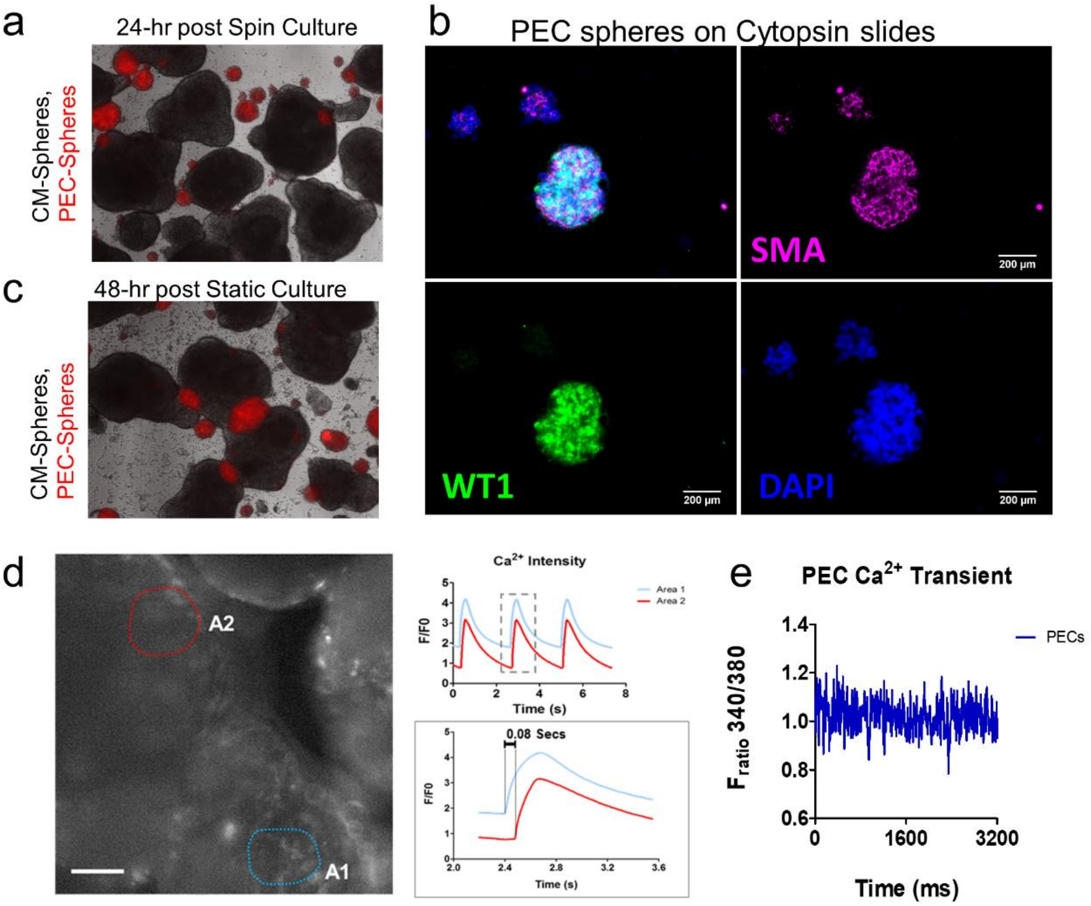

Supplementary Figure 5: Characterization of PEC spheres.
a CM spheres and PEC spheres in spin culture after 24-hr. **b** PEC spheres, generated in spinner flask using PEC Medium with RPMI complete medium in a 1:1 ratio for 24 h, were collected and immuno-stained for SMA (magenta) and WT1 (green) after cytospin and fixation on microscopic slide. CM-spheres and PEC-spheres in static culture after 48 h. Samples were collected and imaged from three independent experiments. **c** CM spheres and PEC spheres in static culture after 48-hr. **d** Representative calcium imaging analysis of a PEC-CM aggregate after 96 hours in co-culture, showing repeated calcium wave propagations from area 1 (A1) to area 2 (A2). Boxed inset enlarged to show Ca^{2+} signal delay. Multiple areas were sampled from 3 independent experiments. Scale, 100 µm. **e** PEC Calcium transient. Day 7 PEC-only cultures were treated with Fura-2 dye, electrically paced (20 V, 0.6 ms, 0.7 Hz), and analyzed under the same methods used to test calcium transients of CM-only and PEC-CM co-culture groups. PECs did not demonstrate a calcium transient flux, suggesting that they did not actively contribute to the calcium transient differences observed between CM-only and PEC-CM cultures. This is a representative reading from 1 of 3 independent experiments.

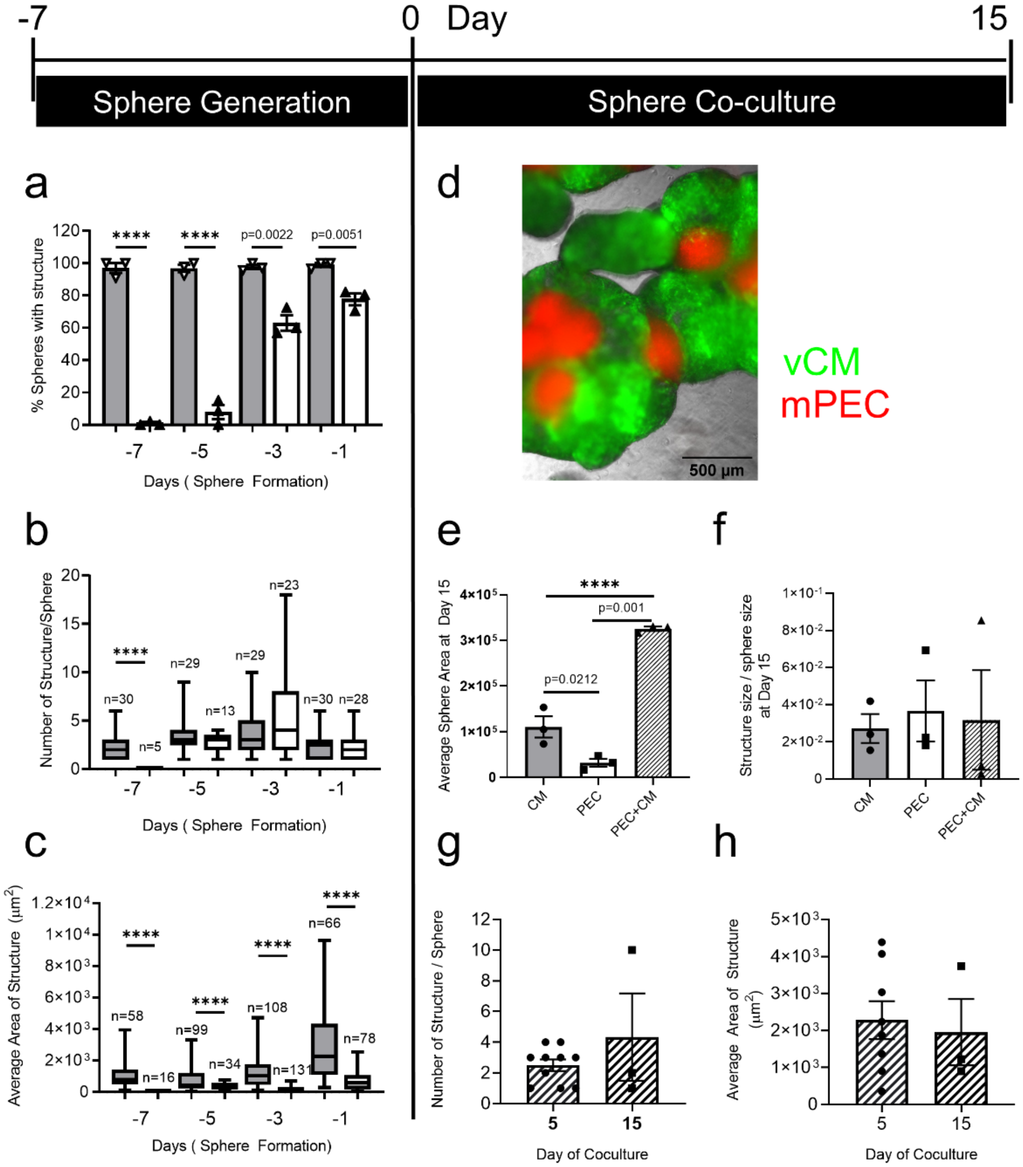

Supplementary Figure 6: Sphere-structure analysis for CM-spheres, and PEC-spheres, PEC-CM aggregates (n=3, each group)

a Percent of spheres with structures over time during sphere formation (bar graph presented mean ± SEM, n= 3 independent experiments, analyzed by two-tailed student T test comparing within CM vs PEC spheres at each recorded day), **b** number of structures per sphere during sphere formation **c** average cross-sectional area of the luminal structures.(data presented box and whisker's plot, with lines indicate 25th, 50th and 75th percentiles and minima and maxima. Unpaired t test with Welch's correction was used. N number presented were pooled CM or PEC spheres over three independent experiments). **d-i** Day 15 analysis: **d** a representative fluorescent image of a PEC-CM aggregate (scale, 500 μm), **e** sphere density, **f** average sphere area, **g** structure size/sphere size (day 5: n= 10 over 3 independent replicates; day 15: n= 3 independent replicates), and **h** average structure cross-sectional area (day 5: n= 9 over 3 independent replicates; day 15: n= 3 independent replicates). **e-h** bar graph presents mean ± SEM. Statistical analysis was performed using one-way ANOVA with Tukey's multiple comparisons test. **** indicates p<0.0001.

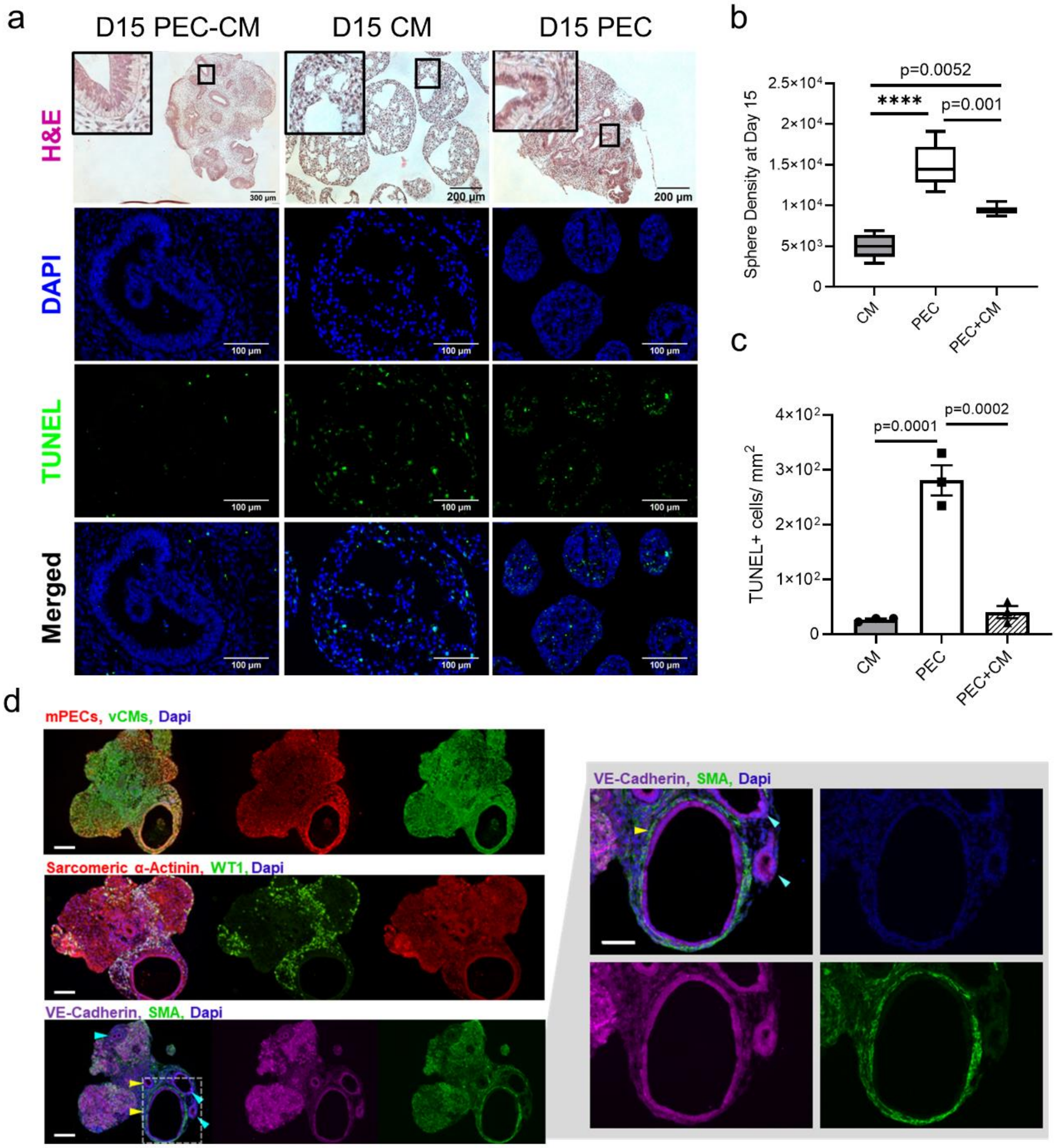

Supplementary Figure 7: PEC-CM co-culture in three-dimensional tissue format.
a Histology of cross-sections from Day 15 PEC-CM aggregates, CM-spheres, and PEC-spheres (n=3); including H&E representations above TUNEL staining analysis from three independent experiments. Scales indicated. **b** Quantification of sphere density (n=10, n=10 and n= 3 for CM, PEC and PEC+CM groups respectively analyzed over three independent replicates). Data presented in box and whiskers plots represent the maxima, 75th percentile, median, 25th percentile and minima.**c** Quantification of TUNEL positive cells in day 15 PEC-CM aggregates, CM-spheres, and PEC-spheres (n=3 independent replicates for each group). Data presented in mean ± SEM. Statistical analysis was performed using one-way ANOVA with Tukey's multiple comparisons test. **d** vCM-spheres and mPEC-spheres integrate to form cardiac micro-tissues with cellular complexity, demonstrating PEC-derived SMA+ and VE-Cadherin+ cells that form higher-order luminal structures, either lined with VE-cadherin+ cells (blue arrows), or VE-cadherin+ cells layered with SMA+ cells (gold arrows). Figure and inset scales, both 100 µm. n=3, Scale bar = 200 µm. Images were obtained from three independent experiments.

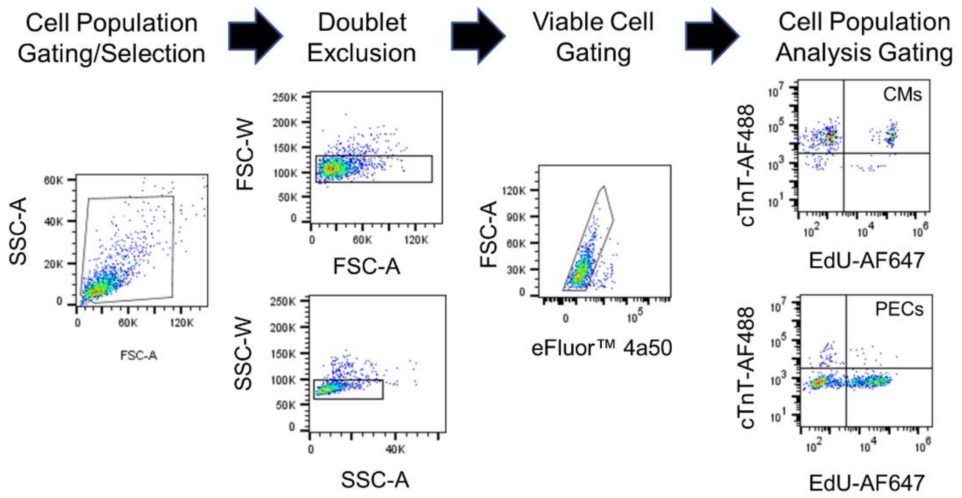

Supplementary Figure 8: Sequential FACS gating strategy.
Dotplot of flow cytometric analysis and gating (left to right) from selection of cell population, doublet exclusion, cell viability-guided selection of target population (eFluor450 staining) and the final fluorescence gating for analyses of, EdU-AF647, cTnT^{+}-AF488 CMs. This gating sequence was used for all flow cytometric analyses presented in this manuscript.

Supplementary Table 1: List of Taqman Probes used in all qPCR experiments.

Taqman Probe Targets	Catalogue number	Manufacturer
WT1	(Cat#: Hs01103751_m1),	ThermoFisher Scientific
TBX18	(Cat#: Hs01385457_m1),	ThermoFisher Scientific
TCF21	(Cat#: Hs00162646_m1),	ThermoFisher Scientific
ALDH1A2	(Cat#: Hs00180254_m1),	ThermoFisher Scientific
UPK1B	(Cat#: Hs01041715_m1),	ThermoFisher Scientific
ATP2A2	(Cat#: Hs00544877_m1),	ThermoFisher Scientific
GJA1	(Cat#: Hs00748445_s1),	ThermoFisher Scientific
ITGA4	(Cat#: Hs00168433_m1)	ThermoFisher Scientific
TNNI3	(Cat#: Hs00165957_m1),	ThermoFisher Scientific
Oct4	(Cat#: Hs00999632_g1),	ThermoFisher Scientific
Sox2	(Cat#: Hs04407947_ml),	ThermoFisher Scientific
Nanog	(Cat#: Hs04260366_g1),	ThermoFisher Scientific
MIXL1	(Cat#: Hs00430824_g1),	ThermoFisher Scientific
T	(Cat#: Hs00610080_m1),	ThermoFisher Scientific
ISL1	(Cat#: Hs0015126_m1),	ThermoFisher Scientific
GATA4	(Cat#:Hs00171403_ml),	ThermoFisher Scientific
Nkx2.5	(Cat#:Hs00231763_m1),	ThermoFisher Scientific
TBX5	(Cat#:Hs00361155_m1),	ThermoFisher Scientific
CDH1	(Cat#:Hs01023894_m1),	ThermoFisher Scientific
CDH2	(Cat#:Hs00983056_m1),	ThermoFisher Scientific
FOXF1	(Cat#:Hs00230962_m1),	ThermoFisher Scientific
PITX2	(Cat#:Hs04234069_mH),	ThermoFisher Scientific
SEMA3D	(Cat#:Hs00380877_m1),	ThermoFisher Scientific
SCX	(Cat#:Hs03054634_g1),	ThermoFisher Scientific
POSTN	(Cat#:Hs01566750_m1),	ThermoFisher Scientific
TGLN2	(Cat#:Hs00761239_s1),	ThermoFisher Scientific
VIM	(Cat#:Hs00185584_m1),	ThermoFisher Scientific
DDR2	(Cat#:Hs01025957_m1),	ThermoFisher Scientific
IGF2	(Cat#:Hs04188276_m1),	ThermoFisher Scientific
MYH6	(Cat#:Hs01101425_m1),	ThermoFisher Scientific
MYL7	(Cat#:Hs01085598_g1),	ThermoFisher Scientific
MYH7	(Cat#:Hs01110632_m1),	ThermoFisher Scientific
MYL2	(Cat#:Hs00166405_m1),	ThermoFisher Scientific
TNNT2	(Cat#:Hs00943911_m1).	ThermoFisher Scientific

Supplementary Table 2: List of Primary antibodies used in the experiment.

Primary antibody	Catalogue number	Application, dilution
Mouse anti-ZO1	Thermo Fisher Scientific Cat# MA339100A488, RRID:AB_2633345	ICC, 1:200
Mouse anti TBX18	R and D Systems Cat# MAB63371, RRID:AB_10892533	ICC, 1:200
Mouse anti-CD31	Agilent Cat# M0823, RRID:AB_2114471	ICC, 1:200
Mouse anti-smooth muscle actin	Abcam Cat# ab7817, RRID:AB_262054	ICC, 1:500
Mouse anti-cardiac troponin T	Abcam Cat# ab8295, RRID:AB_306445	ICC/FC, 1:500
Mouse anti sarcomeric α-actinin	Sigma-Aldrich Cat# A7811, RRID:AB_476766	ICC, 1:250
Mouse anti MLC2A	Abcam Cat# ab68086, RRID:AB_1140497	ICC, 1:200
Rabbit anti-WT1	Abcam Cat# ab89901, RRID:AB_2043201	ICC/FC, 1:250
Rabbit mAb to TCF21	Abcam Cat#ab182134 RRID:AB_2889038	ICC, 1:200
Rabbit anti-RFP	Abcam Cat# ab62341, RRID:AB_945213	ICC, 1:200)
Rabbit anti-GFP	Abcam Cat# ab290, RRID:AB_303395	(1:200)
Rabbit anti-VE-Cadherin	Abcam Cat# ab33168, RRID:AB_870662	(1:200)
Rabbit anti-Calponin	Abcam Cat# ab46794, RRID:AB_2291941	(1:200)
Rabbit anti-MYL2	Abcam Cat# ab92721, RRID:AB_10563535	(1:200)
PE-mouse anti-KDR	BD Biosciences Cat# 560872, RRID:AB_10564096	(FC, 1:50)
Alexa Fluor® 647 anti-mouse/human CD324 (E-Cadherin) Antibody	BioLegend Cat# 147308, RRID:AB_2563955	(1:200)
Alexa Fluor® 647 Mouse Anti-Human CD144 Clone 55-7H1 (RUO)	BD Biosciences Cat# 561567, RRID:AB_10712766	(FC, 1:20)
