# Supplementary material for: Human iPS-derived pre-epicardial cells direct cardiomyocyte aggregation expansion and organization in vitro
Source: Nat Commun. 2021 Aug 17;12:4997. doi: 10.1038/s41467-021-24921-z (PMC8370973; doi:10.1038/s41467-021-24921-z)
Supplement: Supplementary file 3 — Reporting Summary [file 41467_2021_24921_MOESM3_ESM.pdf]

nature research

Corresponding author(s): Harald C Ott, Jun Jie Tan

Last updated by author(s): 6/13/2021

Reporting Summary

Nature Research wishes to improve the reproducibility of the work that we publish. This form provides structure for consistency and transparency in reporting. For further information on Nature Research policies, see our Editorial Policies and the Editorial Policy Checklist.

Statistics

For all statistical analyses, confirm that the following items are present in the figure legend, table legend, main text, or Methods section.

n/a	Confirmed	
☐	☒	The exact sample size (*n*) for each experimental group/condition, given as a discrete number and unit of measurement
☐	☒	A statement on whether measurements were taken from distinct samples or whether the same sample was measured repeatedly
☐	☒	The statistical test(s) used AND whether they are one- or two-sided<br>*Only common tests should be described solely by name; describe more complex techniques in the Methods section.*
☒	☐	A description of all covariates tested
☐	☒	A description of any assumptions or corrections, such as tests of normality and adjustment for multiple comparisons
☐	☒	A full description of the statistical parameters including central tendency (e.g. means) or other basic estimates (e.g. regression coefficient) AND variation (e.g. standard deviation) or associated estimates of uncertainty (e.g. confidence intervals)
☐	☒	For null hypothesis testing, the test statistic (e.g. *F*, *t*, *r*) with confidence intervals, effect sizes, degrees of freedom and *P* value noted<br>*Give P values as exact values whenever suitable.*
☒	☐	For Bayesian analysis, information on the choice of priors and Markov chain Monte Carlo settings
☒	☐	For hierarchical and complex designs, identification of the appropriate level for tests and full reporting of outcomes
☒	☐	Estimates of effect sizes (e.g. Cohen's *d*, Pearson's *r*), indicating how they were calculated

Our web collection on statistics for biologists contains articles on many of the points above.

Software and code

Policy information about availability of computer code

Data collection

Sarcomere length and cell size were measured using FIJI/ImageJ (ImageJ 1.53c, NIH). The sequencing data were uploaded to the Galaxy web platform and were pre-processed at the public server at usegalaxy.org. Fastq files were read and trimmed by using Trimmomatic. First 10 bases were clipped and sequences below the average Phred score of 30 within a sliding window of 4 bases were trimmed. Results from individual samples were aggregated using MultiQC .Sequences from passed samples were mapped to hg38 human reference genome using Burrows-Wheeler Alignment tool for short sequences (<100bp). Generated BAM files were merged accordingly and subsequently used to produce gene counts using featureCounts v1.6.4 based on simple Illumina analysis mode. We used iDEP.90, an integrated web application for differential gene expression and functional ontology analyses. A pseudo count of 4 were added to the gene count before edgeR Bioconductor package was used to transform counts data (log2(CPM+4)) for clustering and principal component analysis.

Area strain analysis was done by performing High Density Mapping (HDM) - a technique that we've previously used in both in vitro and in vivo cardiac analyses to determine micron-level displacements within cardiomyocytes and cardiac tissues, first described by Kelly et al (Medical Engineering & Physics, 29. 2007. p 154–162). High contrast digital grayscale images of a surface undergoing deformation are acquired. After image capture, the region of interest (ROI) in consecutive images are segmented into small image fields, each named a subimage, for individual cross-correlation. image registration is performed on multiple subimages, each corresponding to a different location on the cardiac field (or contracting area), which allows the determination of a 2D displacement vector matrix over the ROI. The unique light intensity distributions for each subimage in both the undeformed and deformed states are used todetermine the x0 and y0 displacements along the x- and y-axes, respectively. The use of phase correlation to determine displacement has been well described: Briefly, discrete Fourier transforms (DFTs) are used to transform the characteristic intensity distributions of subimages from the spatial domain to the spectral domain, where a unique phase shift corresponds to a displacement in the spatial domain. The independent spectra from the undeformed and deformed images, are correlated with a cross-power spectrum. the impulse function obtained by the normalized cross-power spectrum is a downsampled Dirichlet kernel that is approximated as a sinc function. This sinc function is interpolated to determine the subpixel motion across subimages. Multiple consecutive subimages are analyzed over the course of each myocyte contraction to determine the full range of deformation, and then multiple contractions are analyzed to determine average area strain within the ROI.

Data analysis	Images were analyzed using FIJI/ImageJ (1.53c, NIH).<br>Flow cytometric data were analyzed using FlowJo 10.5.0 software.<br>Graphs were generated using GraphPad Prism 8.0.2. software.

For manuscripts utilizing custom algorithms or software that are central to the research but not yet described in published literature, software must be made available to editors and reviewers. We strongly encourage code deposition in a community repository (e.g. GitHub). See the Nature Research guidelines for submitting code & software for further information.

Data

Policy information about availability of data

All manuscripts must include a data availability statement. This statement should provide the following information, where applicable:

- Accession codes, unique identifiers, or web links for publicly available datasets
- A list of figures that have associated raw data
- A description of any restrictions on data availability

The RNA sequencing data discussed in this publication have been deposited in NCBI's Gene Expression Omnibus 66 and are accessible through GEO Series accession number GSE148543 https://www.ncbi.nlm.nih.gov/geo/query/acc.cgi?acc=GSE148543. Data that support the findings of this study are available from the corresponding authors upon reasonable request.

Field-specific reporting

Please select the one below that is the best fit for your research. If you are not sure, read the appropriate sections before making your selection.

[x] Life sciences [ ] Behavioural & social sciences [ ] Ecological, evolutionary & environmental sciences

For a reference copy of the document with all sections, see nature.com/documents/nr-reporting-summary-flat.pdf

Life sciences study design

All studies must disclose on these points even when the disclosure is negative.

Sample size	We did not perform sample size calculation for power on hypotheses in this study. We decided on the sample size based on previous literatures which has shown to be sufficient for the analysis of statistical significance. All the in vitro experiments were repeated independently for at least in 3 times unless stated otherwise in the manuscript.
Data exclusions	No data exclusion is performed in data collection and analysis.
Replication	All experiments were performed in at least three independent replicates and the number of independent repeat for each findings is included in the description of the figure legends. All attempts at replication were successful.
Randomization	All cultured cell used for experimental and control groups were randomly selected and assigned.
Blinding	Experiment design did not need blinding as the findings could not be influenced by the investigators

Reporting for specific materials, systems and methods

We require information from authors about some types of materials, experimental systems and methods used in many studies. Here, indicate whether each material, system or method listed is relevant to your study. If you are not sure if a list item applies to your research, read the appropriate section before selecting a response.

Materials & experimental systems

n/a	Involved in the study	
[ ]	[x]	Antibodies
[ ]	[x]	Eukaryotic cell lines
[x]	[ ]	Palaeontology and archaeology
[x]	[ ]	Animals and other organisms
[x]	[ ]	Human research participants
[x]	[ ]	Clinical data
[x]	[ ]	Dual use research of concern

Methods

n/a	Involved in the study	
[x]	[ ]	ChIP-seq
[ ]	[x]	Flow cytometry
[x]	[ ]	MRI-based neuroimaging

Antibodies

Antibodies used	Mouse anti-ZO1 Monoclonal Antibody, Thermo Fisher Scientific Cat# MA339100A488, RRID:AB_2633345 ICC, 1:200<br>Mouse anti TBX18 Monoclonal Antibody, R and D Systems Cat# MAB63371, RRID:AB_10892533 ICC, 1:200<br>Mouse anti-CD31 Monoclonal Antibody, Agilent Cat# M0823, RRID:AB_2114471 ICC, 1:200

Mouse anti-Smooth Muscle Actin Monoclonal Antibody, Abcam Cat# ab7817, RRID:AB_262054 ICC, 1:500
Mouse anti-Cardiac Troponin T Monoclonal Antibody, Abcam Cat# ab8295, RRID:AB_306445 ICC/FC, 1:500
Mouse anti Sarcomeric α-actinin Monoclonal Antibody, Sigma-Aldrich Cat# A7811, RRID:AB_476766 ICC, 1:250
Mouse anti MLC2A Polyclonal Antibody, Abcam Cat# ab68086, RRID:AB_1140497 ICC, 1:200
Rabbit anti-WT1 Monoclonal Antibody, Abcam Cat# ab89901, RRID:AB_2043201 ICC/FC, 1:250
Rabbit anti-TCF21 Monoclonal Antibody, Abcam Cat#ab182134 RRID:AB_2889038 ICC, 1:200
Rabbit anti-RFP Polyclonal Antibody, Abcam Cat# ab62341, RRID:AB_945213 ICC, 1:200)
Rabbit anti-GFP Polyclonal Antibody, Abcam Cat# ab290, RRID:AB_303395 (1:200)
Rabbit anti-VE-Cadherin Polyclonal Antibody, Abcam Cat# ab33168, RRID:AB_870662 (1:200)
Rabbit anti-Calponin Monoclonal Antibody, Abcam Cat# ab46794, RRID:AB_2291941 (1:200)
Rabbit anti-MYL2 Monoclonal Antibody, Abcam Cat# 92721, RRID:AB_10563535 (1:200)
PE-mouse anti-KDR Monoclonal antibody BD Biosciences Cat# 560872, RRID:AB_10564096 (FC, 1:50)
Alexa Fluor® 647 anti-mouse/human CD324 (E-Cadherin) Monoclonal Antibody, BioLegend Cat# 147308, RRID:AB_2563955 (1:200)
Alexa Fluor® 647 Mouse Anti-Human CD144 Monoclonal antibody Clone 55-7H1 (RUO) BD Biosciences Cat# 561567, RRID:AB_10712766 (FC, 1:20)

Validation

Mouse anti-ZO1 Monoclonal Antibody, Thermo Fisher Scientific was validated using Caco-2 cells, as provided on the manufacturer's website.

Mouse anti TBX18 Monoclonal Antibody, R and D Systems TBX18 was detected in immersion fixed Saos-2 human osteosarcoma cell line, as provided on the manufacturer's website

Mouse anti-CD31 Monoclonal Antibody was validated using human umbilical vascular endothelial cells (HUVECs, Supplementary figure 1)

Mouse anti-Smooth Muscle Actin Monoclonal Antibody, Abcam Cat# ab7817, ab7817 has been referenced in 610 publications, as shown on the manufacturer's website.

Mouse anti-Cardiac Troponin T Monoclonal Antibody, Abcam Cat# ab8295, ab8295 has been referenced in 153 publications, as shown on the manufacturer's website.

Mouse anti Sarcomeric α-actinin Monoclonal Antibody, Sigma-Aldrich Cat# A7811, validated by the manufacturer using human cardiac muscle or tongue, as shown on the manufacturer's website.

Mouse anti-MLC2A Polyclonal Antibody, Abcam Cat# ab68086 has been referenced in 4 publications, as shown on the manufacturer's website.

Rabbit anti-WT1 Monoclonal Antibody, Abcam Cat# ab89901 has been referenced in 115 publications, as shown on the manufacturer's website.

Rabbit anti-TCF21 Monoclonal Antibody, Abcam Cat#ab182134 was validated using HeLa cells, as provided on the manufacturer's website.

Rabbit anti-RFP Polyclonal Antibody, Abcam Cat# ab62341 has been referenced in 199 publications, as shown on the manufacturer's website.

Rabbit anti-GFP Polyclonal Antibody, Abcam Cat# ab290 has been referenced in 2351 publications, as shown on the manufacturer's website.

Rabbit anti-VE-Cadherin Polyclonal Antibody, Abcam Cat# ab33168 has been referenced in 195 publications, as shown on the manufacturer's website.

Rabbit anti-Calponin Monoclonal Antibody, Abcam Cat# ab46794, ab46794 has been referenced in 178 publications, as shown on the manufacturer's website.

Rabbit anti-MYL2 Monoclonal Antibody, Abcam Cat# 92721, ab92721 has been referenced in 26 publications, as shown on the manufacturer's website.

Alexa Fluor® 647 anti-mouse/human CD324 (E-Cadherin) Monoclonal Antibody, BioLegend Cat# 147308, has been referenced in 7 publications, as shown on the manufacturer's website.

PE-mouse anti-KDR Monoclonal antibody BD Biosciences Cat# 560872, and Alexa Fluor® 647 Mouse Anti-Human CD144 Monoclonal antibody Clone 55-7H1 (RUO) BD Biosciences Cat# 561567 were validated using HUVECs as provided on the manufacturer's website.

Eukaryotic cell lines

Policy information about cell lines

Cell line source(s)

BJR induced-pluripotent stem cells derived from human, male newborn fibroblasts (Developed by the Harvard Stem Cell Institute iPS Cell Core Facility; developed by inhibitory mRNA induction of the BJ (ATCC CRL-2522)-HS human fibroblast line,

	purchased from ATCC); the Gibco hiPSC Episomal iPSC line reprogrammed from cord blood (Gibco, Grand Island, NY); ATCC DYS0100 hiPSCs (ATCC ACS-1019) reprogrammed from foreskin fibroblast (ATCC, Manassas, VA); and H9-hESCs-derived epicardial cells (established and provided by Palecek's lab).
Authentication	All lines were obtained from the providers. no authentication was performed
Mycoplasma contamination	All lines were tested with mycoplasma and were found negative.
Commonly misidentified lines (See ICLAC register)	None of the cell lines are listed in ICLAC database.

Flow Cytometry

Plots

Confirm that:

☒ The axis labels state the marker and fluorochrome used (e.g. CD4-FITC).

☒ The axis scales are clearly visible. Include numbers along axes only for bottom left plot of group (a 'group' is an analysis of identical markers).

☒ All plots are contour plots with outliers or pseudocolor plots.

☒ A numerical value for number of cells or percentage (with statistics) is provided.

Methodology

Sample preparation	Cells were dissociated using trypsin, pelleted by centrifugation at 300 g for 5 min, and fixed with Fixation/Permeabilization Solution Kit according to manufacturer's protocol (BD Biosciences, San Diego, CA). Cells were washed with 2 times of 10% BD Perm/Wash Buffer (BD Biosciences, San Diego, CA) to remove fixative prior to staining. Samples were incubated with primary antibody diluted in BD Perm/Wash Buffer (see Online Table I for dilutions) at 4oC for 45 min. To remove excessive primary antibody, samples were spun at 250 g for 5 min and washed once with Perm/Wash buffer prior to labelling with Alexa Fluor 488 secondary antibody (Molecular Probes Eugene, OR) at 1:500 for 30 min.
Instrument	BD Accuri, BD FACS LSR Flow Cytometer (BD Biosciences, San Diego, CA) or NovoCyte Flow Cytometer (ACEA Biosciences, San Diego, CA).
Software	Analysis for flow cytometry was performed with FlowJo VX software version 9.9.4.
Cell population abundance	Derivation of proepicardial-like cells from BJRiPSCs using the method described in the manuscript yielded about 86.8% WT1+ cells at day 7; whereas derivation of cardiomyocytes from the same day 3 lateral plate mesodermal source from BJRiPSCs achieved 81% cTnT+ cells.
Gating strategy	iPSC-derived cardiomyocytes and iPSC-derived preepicardial-like cells were used to gate FSC/SSC, and to exclude doublets. Negative controls were samples that stained with secondary only or with IgG isotype. Positive cells were gated based on dotplot.

☒ Tick this box to confirm that a figure exemplifying the gating strategy is provided in the Supplementary Information.
