# Supplementary material for: Human iPS-derived pre-epicardial cells direct cardiomyocyte aggregation expansion and organization in vitro
Source: Nat Commun. 2021 Aug 17;12:4997. doi: 10.1038/s41467-021-24921-z (PMC8370973; doi:10.1038/s41467-021-24921-z)
Supplement: Supplementary file 4 — Description of Additional Supplementary Files [file 41467_2021_24921_MOESM4_ESM.pdf]

Description of Additional Supplementary Files

Supplementary Movie 1
Representative movie recording of contracting venus-expressing CM in PEC/CM co-differentiation culture from three independent experiments.

Supplementary Movie 2
Representative movie recording of CMs in CM-only culture at day 7, contracting under paced conditions (20 V, 0.6 ms, 0.7 Hz) from three independent experiments.

Supplementary Movie 3
Representative movie recording of CMs in PEC-CM co-culture at day 7, contracting under paced conditions (20 V, 0.6 ms, 0.7 Hz) from three independent experiments.

Supplementary Movie 4
Representative movie of elongated, contracting ventricular-like myocytes differentiated from iPS cells after 96h RXR inhibition using BMS-189453 from three independent experiments.

Supplementary Movie 5
Representative movie of compacted ventricular-like myocytes form contracting network in PEC coculture, from three independent experiments.

Supplementary Movie 6
Representative movie of spontaneously contracting day 15 CM-spheres, demonstrating independent electromechanical function, from three independent experiments.

Supplementary Movie 7
Representative movie of spontaneously contracting PEC-CM aggregates, demonstrating propagation of [Ca2+] transients and coupled electromechanical activity, from three independent experiments.
